# Application of Teach-back health education combined with continuity care in patients with knee joint training injuries: an analysis of clinical effects

**DOI:** 10.3389/fpubh.2025.1577538

**Published:** 2025-05-21

**Authors:** Huijun Li, Wei Li, Xiangjing Kong, Zhaodi Wang, Conglan Lu, Dongqin Shi

**Affiliations:** ^1^Nursing Department, Air Force Hospital of Eastern Theater, Nanjing, Jiangsu, China; ^2^Orthopedics Department, Air Force Hospital of Eastern Theater, Nanjing, Jiangsu, China

**Keywords:** Teach-back, continuity care, knee joint training injury, health education, nursing

## Abstract

**Objective:**

To investigate the clinical efficacy of Teach-back health education combined with continuity care in patients with knee joint training injuries.

**Methods:**

A total of 210 patients with knee joint training injuries admitted to our hospital from May 2023 to August 2024 were randomly divided into an observation group and a control group (105 cases each) using a random number table. The control group received routine care, while the observation group received Teach-Back health education combined with continuity care. Outcomes, including the Hospital for Special Surgery (HSS) knee score, Generic Quality of Life Inventory-74 (GQOLI-74), Health-Promoting Lifestyle Profile-II (HPLP-II), State-Trait Anxiety Inventory (STAI) scores, and postoperative complication rates, were compared between the two groups before and 2 months after intervention.

**Results:**

At 2 months post-intervention, both groups showed significant improvements in HSS, GQOLI-74, and HPLP-II scores compared to baseline (*P* < 0.05), with the observation group outperforming the control group (*P* < 0.05). At 2 weeks post-intervention, the observation group exhibited lower STAI scores than the control group (*P* < 0.05). The incidence of complications (deep vein thrombosis, joint stiffness, pressure injuries, and infections) was significantly lower in the observation group (*P* < 0.05).

**Conclusion:**

Teach-Back health education combined with continuity care effectively promotes functional recovery, improves quality of life and psychological wellbeing, enhances health-promoting behaviors, and reduces postoperative complications in patients with knee joint training injuries. This approach holds significant clinical value for accelerating rehabilitation and maintaining military readiness.

## 1 Introduction

Patient education serves as a cornerstone of effective rehabilitation, particularly in military or athletic populations prone to training-related injuries. Among diverse pedagogical approaches, the Teach-back method (also termed “feedback education”) has emerged as a validated health literacy intervention (1, 2). This bidirectional communication strategy requires learners to verbally restate received health information, enabling educators to immediately assess comprehension accuracy, and address knowledge gaps through iterative clarification cycles. The four-phase Teach-back framework—knowledge delivery, information feedback, content clarification, and knowledge consolidation—adopts a “theory-practice-theory” spiral learning model that synergizes cognitive understanding with practical application. When integrated with continuity of care systems, this methodology may substantially enhance long-term rehabilitation outcomes.

Continuity of care, extending institutional nursing services to community settings through digital platforms and home visits, addresses the critical transition from hospital-based to self-managed care. Its implementation proves particularly crucial for joint injuries requiring sustained rehabilitation. Epidemiological analyses reveal that lower extremity training injuries predominantly affect the knee joint (3). The knee's susceptibility stems from its complex multiaxial biomechanics: as a polyarticular hinge joint facilitating multiplanar movements, it sustains tremendous shear forces during high-intensity training. Moreover, the avascular nature of the meniscus severely limits its self-repair capacity. Cumulative microtrauma from improper loading patterns or repetitive kinematic errors frequently progresses to meniscal degeneration or full-thickness tears, often culminating in chronic joint dysfunction if unaddressed.

Current rehabilitation paradigms face dual challenges: (1) insufficient patient health literacy regarding biomechanical principles and home exercise protocols, and (2) discontinuity between clinical supervision and self-management phases (4). Traditional didactic education often fails to ensure knowledge retention, while conventional discharge planning lacks mechanisms for continuous adherence monitoring. This gap becomes particularly detrimental for knee injury patients requiring precise long-term compliance with weight-bearing restrictions and neuromuscular re-education exercises.

We hypothesize that integrating Teach-back's active learning framework with continuity of care technologies could create a synergistic intervention model. By establishing closed-loop feedback channels between clinicians and patients, this approach may achieve three critical objectives: (1) enhance comprehension of injury mechanisms through iterative knowledge verification, (2) maintain therapeutic exercise fidelity via remote supervision, and (3) facilitate early detection of movement compensation patterns predisposing to reinjury. To test this premise, we designed a prospective cohort study evaluating the feasibility and efficacy of combined Teach-back/continuity of care interventions in military personnel with knee training injuries. Through quantitative analysis of functional recovery metrics and qualitative assessment of health behavior modification, this investigation aims to elucidate the intervention's mechanistic pathways and optimize rehabilitation protocols for complex joint injuries.

## 2 Materials and methods

### 2.1 General information

A total of 210 patients with knee joint training injuries admitted to our hospital from May 2023 to August 2024 were randomly allocated using a random number table method into two groups: an observation group and a control group, each comprising 105 patients. The control group consisted of 53 ligament injuries, 28 meniscus injuries, 12 cases of chondromalacia patellae, 5 knee synovitis cases, and 7 fractures. Educational backgrounds included 73 high school graduates and 32 college graduates, with ages ranging from 18 to 26 years (mean age 22.12 ± 2.35). The observation group included 58 ligament injuries, 35 meniscus injuries, 11 cases of chondromalacia patellae, 5 knee synovitis cases, and 6 fractures. Educational backgrounds comprised 74 high school graduates and 41 college graduates, with ages ranging from 17 to 26 years (mean age 21.32 ± 2.25). Comparative analysis revealed no significant differences between the two groups in terms of injury types, age distribution, or educational background (*P* > 0.05; Table 1).

**Table 1:** Baseline characteristics of patients.

**Characteristic**	**Observation group (*n* = 105)**	**Control group (*n* = 105)**	***P-value***
Age (years), mean ± SD	21.32 ± 2.25	22.12 ± 2.35	>0.05
**Education level**, ***n*** **(%)**			
- High school	74 (70.48%)	73 (69.52%)	>0.05
- College	41 (39.05%)	32 (30.48%)	>0.05
**Diagnosis**, ***n*** **(%)**			
- Ligament injury	58 (55.24%)	53 (50.48%)	>0.05
- Meniscus injury	35 (33.33%)	28 (26.67%)	>0.05
- Patellar chondromalacia	11 (10.48%)	12 (11.43%)	>0.05
- Knee synovitis	5 (4.76%)	5 (4.76%)	>0.05
- Fracture	6 (5.71%)	7 (6.67%)	>0.05

Data presented as mean ± SD or n (%). *P*-values indicate no significant differences between groups (*P* > 0.05).

### 2.2 Inclusion and exclusion criteria

Inclusion criteria: (a) Male recruits enlisted within the past 2 years. (b) Patients undergoing surgical intervention for exercise-induced knee injuries sustained within 6–12 months. (c) Demonstrated compliance and voluntary participation in health education. (d) Non-smokers and alcohol abstinence.

Exclusion criteria: (a) Service duration exceeding 2 years. (b) Injuries persisting beyond 12 months prior to enrollment. (c) Poor adherence to study protocols. (d) Female participants.

### 2.3 Implementation method

The control group received routine care, including enhanced condition monitoring, health education, and rehabilitation guidance during hospitalization. After discharge, one telephone follow-up was conducted to inquire about patients' physical status and provide corresponding guidance and assistance. The observation group received Teach-back health education combined with extended care, implemented as follows.

#### 2.3.1 Establishment of a Teach-back health education and extended care team

A multidisciplinary team (MDT) comprising 7 members was formed: 2 orthopedic surgeons, 1 psychologist, 2 orthopedic specialist nurses, 1 rehabilitation therapist, and 1 orthopedic outpatient nurse. All members demonstrated strong communication, coordination, and expressive skills, with expertise in their respective fields. Responsibilities were clearly defined to ensure interdisciplinary collaboration.

#### 2.3.2 Implementation of Teach-back health education

Orthopedic specialist nurses scientifically developed a knowledge assessment questionnaire on rehabilitation care for patients with knee training injuries (Supplementary Table 1) and implemented the Teach-back method for each questionnaire item during health education sessions. The process included four steps— Explanation: for each educational session, nurses focused on patients' knowledge gaps, addressing 3–5 key points within 30 min to optimize comprehension. Educational formats included verbal instruction, videos, and health education materials, scheduled during patients' free time. Restatement: patients were asked to paraphrase the information using pre-designed questions. Evaluation: nurses assessed patients' restatements. Incomplete or inaccurate responses prompted targeted re-education until mastery was achieved. Communication techniques were employed to minimize psychological pressure for patients with poor comprehension. Comprehension: open-ended questions were used to evaluate understanding of accelerated rehabilitation for training-induced knee injuries. A health record was established for discharged patients, supplemented by a WeChat group for sharing postoperative rehabilitation knowledge, monitoring compliance, and facilitating peer interaction.

#### 2.3.3 Perioperative care optimization for knee training injuries

Preoperative guidance: upon admission, patients, and families received education on knee injury etiology, surgical procedures, and postoperative rehabilitation through age- and education-appropriate materials (e.g., videos, pamphlets). Nurses assessed psychological status through behavioral observation and communication, providing positive reinforcement and targeted counseling. Fasting protocol adjustment: preoperative fasting was limited to 6 h for solids and 2 h for liquids. Patients with chronic conditions were permitted small amounts of water for morning medications. Bowel preparation was omitted for patients with regular bowel movements. Preoperative intravenous hydration was administered as needed. Postoperative dietary intervention: oral hydration (10 mL) was initiated upon return to the ward if no nausea/vomiting occurred. Swallowing function was assessed before advancing to 50 mL water. Liquid diets began at 4 h postoperatively, transitioning to semi-liquid and regular diets by 6 h to promote early nutritional intake. Pain management: patients were trained to self-assess pain using the Numerical Rating Scale (NRS) and Wong-Baker FACES Pain Rating Scale. Nurses recorded daily pain scores and employed multimodal analgesia, combining non-pharmacological interventions (e.g., deep breathing, massage, distraction, thermal therapy) with pharmacological approaches (NSAIDs, weak/strong opioids). Analgesic pumps were utilized, with pain assessments conducted at least once per shift. Multidisciplinary collaboration with physicians and anesthesiologists ensured optimal pain control, supplemented by emotional support to reduce pain sensitivity. Psychological support: nurses encouraged positive coping strategies through effective communication, tailoring psychological interventions based on patient age, personality, and preferences in collaboration with families. Rehabilitation guidance: structured exercises included muscle strengthening and knee joint mobility training. Given that ligament injuries and meniscus injuries constitute the predominant injury patterns, we have established dedicated rehabilitation protocols for these two conditions (Supplementary methods).

#### 2.3.4 Extended care protocol

Follow-ups were conducted at 1 week, 1 month, 2 months, 3 months, and 6 months post-discharge, covering: (1) Rehabilitation monitoring: wound healing, exercise adherence, self-care capacity, muscle strength, joint mobility, weight-bearing precautions for internal fixation patients, and external fixator stability/skin condition. (2) Dietary guidance: high-protein, high-calcium, and vitamin-rich diets were recommended. Patients with comorbidities received tailored dietary advice, with avoidance of cold, spicy, or indigestible foods and contraindications for medication administration. (3) Psychological reinforcement: patients exhibiting dependency behaviors were encouraged to gradually resume daily activities and social roles. (4) Feedback collection: patient suggestions regarding care quality were documented and relayed to department heads for continuous improvement. (5) Follow-up coordination: re-examination appointments were scheduled, with reminders to bring medical records and discharge summaries.

### 2.4 Outcome measures

#### 2.4.1 Demographic data

Baseline characteristics, including gender, age, educational background, family support, lifestyle habits, specific preferences, disease duration, clinical condition, treatment history, and presence of complications, were compared between the two groups.

#### 2.4.2 Knee function assessment

The Hospital for Special Surgery (HSS) Knee Score was utilized to evaluate knee function (5). This scale comprises seven items, with six scored components (pain, functional capacity, range of motion, muscle strength, flexion deformity, and joint stability) and one deduction component (use of assistive devices, varus/valgus deformity, and extension limitations). Scores were categorized as follows: excellent (>85 points), good (70–84 points), fair (60–69 points), and poor (<59 points). The magnitude of improvement after rehabilitation intervention was defined based on changes in the HSS. Participants were categorized into the responder group if their HSS score increased by ≥5.41 points from baseline to post-intervention. This threshold has been previously validated as the Minimum Clinically Important Difference (MCID) for assessing knee functional recovery (6).

#### 2.4.3 Quality of life evaluation

The Generic Quality of Life Inventory-74 (GQOLI-74) was employed to assess quality of life across four domains: psychological function, physical function, material wellbeing, and social function. Each item was rated on a 1–5 scale, with lower scores indicating poorer quality of life (7).

#### 2.4.4 Psychological status assessment

The State-Trait Anxiety Inventory (STAI) was used to evaluate psychological states (8). This 40-item scale consists of two subscales: State Anxiety (S-AI) and Trait Anxiety (T-AI), with each item scored 1–4 (higher scores indicating poorer psychological status.

#### 2.4.5 Health-promoting behaviors

The Health-Promoting Lifestyle Profile II (HPLP-II) was adopted to assess health-promoting behaviors (9). This 52-item scale covers six dimensions: health responsibility, physical activity, self-actualization, nutrition, interpersonal relationships, and stress management. Each item was rated on a 4-point Likert scale (1 = “never,” 4 = “always”). Total scores ranged from 52 to 208, categorized as excellent (172–208), good (132–171), fair (92–131), and poor (52–91). A mean item score <2.5 indicated suboptimal health-promoting behaviors.

#### 2.4.6 Postoperative complications

Incidence rates of postoperative complications, including nausea/vomiting (6 h and 24 h postoperatively), deep vein thrombosis, pressure injury, joint stiffness, and infection, were compared between the groups.

### 2.5 Statistical analysis

All statistical analyses were performed using SPSS software (version 22.0; IBM Corp.). Continuous variables with normal distribution were expressed as mean ± standard deviation (SD) and analyzed using two-way analysis of variance (ANOVA) as appropriate. Categorical variables were presented as frequencies with percentages [*n* (%)] and compared using Pearson's Chi-square test or Fisher's exact test, depending on expected cell frequencies. Ordinal data were analyzed using non-parametric Mann-Whitney *U*-test or Kruskal-Wallis *H*-test when comparing between two groups or multiple groups, respectively. For all inferential analyses, a two-tailed *P-value* < 0.05 was considered statistically significant. Prior to parametric testing, assumptions of normality were verified using Shapiro-Wilk tests, and homogeneity of variances was assessed using Levene's test. When data violated parametric assumptions, appropriate non-parametric alternatives were employed.

## 3 Results

### 3.1 Comparison of knee function scores between groups

**Figure 1:**
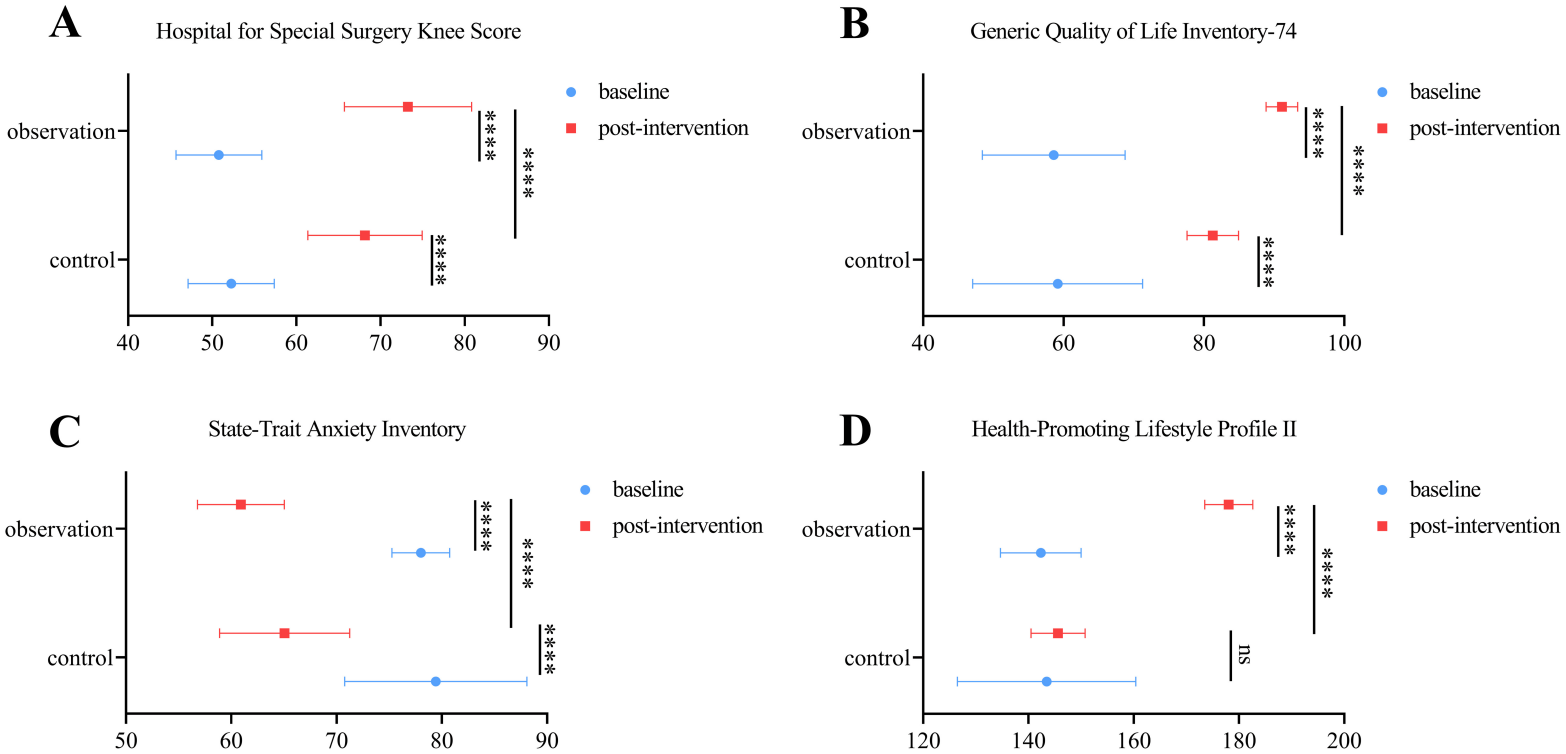
Function assessments. **(A–D)** Comparison of knee function **(A)**, quality of life **(B)**, psychological status **(C)**, and health-promoting behaviors **(D)** assessments in control and observation group at baseline and post-intervention. Results are expressed as means ± SD. ^****^P < 0.0001.

At 2 months post-intervention, both groups showed significant improvements in Hospital for Special Surgery (HSS) compared to baseline (control group: 52.25 ± 5.12 vs. 68.15 ± 6.79, *P* < 0.0001; observation group: 50.79 ± 5.10 vs. 73.26 ± 7.56, *P* < 0.0001). Moreover, the observation group demonstrated significantly higher HHS scores than the control group after intervention (68.15 ± 6.79 vs. 73.26 ± 7.56, *P* < 0.0001, Figure 1A). However, all between-group differences exceeded the MCID.

### 3.2 Comprehensive quality of life assessment

After 2 months, both groups exhibited significant increases in the General Quality of Life Inventory-74 (GQOLI-74) scores compared to baseline (control group: 59.17 ± 12.11 vs. 81.28 ± 3.67, *P* < 0.0001; observation group: 58.61 ± 10.17 vs. 91.12 ± 2.24, *P* < 0.0001). The observation group achieved significantly higher scores than the control group after intervention (81.28 ± 3.67 vs. 91.12 ± 2.24, *P* < 0.0001, Figure 1B).

### 3.3 Psychological status evaluation

At 2 weeks post-intervention, the State-Trait Anxiety Inventory (STAI) scores in the observation group were significantly lower than baseline (78.01 ± 2.74 vs. 60.92 ± 4.12, *P* < 0.0001, Figure 1C) and also significantly lower than those in the control group (65.07 ± 6.18 vs. 60.92 ± 4.12, *P* < 0.0001, Figure 1C).

### 3.4 Health-promoting behaviors

After 2 months, the observation group showed significant improvements in the Health-Promoting Lifestyle Profile II (HPLP-II) scores compared to baseline (142.35 ± 7.65 vs. 178.07 ± 4.58, *P* < 0.0001, Figure 1D), while the control group exhibited no significant change (143.46 ± 16.94 vs. 145.63 ± 5.14, *P* > 0.05, Figure 1D). The observation group also outperformed the control group (145.63 ± 5.14 vs. 178.07 ± 4.58, *P* < 0.0001, Figure 1D).

### 3.5 Comparison of complication rates

Although individual complication rates (e.g., nausea, vomiting, deep vein thrombosis) did not differ significantly between groups (*P* > 0.05), the total complication rate in the observation group was significantly lower than that in the control group (11.43% vs. 30.48%, *P* < 0.001; Table 2).

**Table 2:** Post-intervention complication rates (*n*, %).

**Group**	**Nausea (6 h)**	**Vomiting (24 h)**	**DVT**	**Joint stiffness**	**Pressure injury**	**Infection**	**Total complications**
Observation (*n* = 105)	5 (4.76%)	4 (3.81%)	0 (0%)	2 (1.90%)	0 (0%)	1 (0.95%)	12 (11.43%)
Control (*n* = 105)	7 (6.67%)	6 (5.71%)	4 (3.81%)	7 (6.67%)	3 (2.86%)	5 (4.76%)	32 (30.48%)
*P*-value	>0.05	>0.05	>0.05	>0.05	>0.05	>0.05	<0.001

## 4 Discussion

As a multi-axial joint capable of multi-planar movement, the knee is prone to acute injuries during high-intensity sustained activities. The meniscus, characterized by limited blood supply and poor self-healing capacity, is susceptible to excessive wear or rupture due to chronic improper use or repetitive incorrect movements. It is critical for patients to master rehabilitation knowledge for knee joint training injuries to facilitate recovery and prompt return to military training. Current health education practices fail to meet the clinical needs of hospitalized patients, necessitating enhanced efforts by healthcare providers to improve health literacy, emphasize skill development, and monitor health behaviors, thereby optimizing resource utilization (10). Historical data indicate that 40%−80% of health education information is immediately forgotten post-intervention, with half of retained information being erroneous (11). Thus, tailored educational approaches are essential to enhance information retention among patients with varying health literacy levels. This study implemented Teach-back health education combined with continuity of care for knee training injury patients, aiming to accelerate rehabilitation and provide theoretical support for clinical application.

Joint function serves as a pivotal metric for evaluating nursing quality in knee training injury patients. Our results demonstrated significantly higher knee function scores in the observation group compared to the control group (*P* < 0.05), indicating that the integrated Teach-back and continuity of care approach effectively promotes functional recovery. Of clinical importance, findings from a prospective randomized controlled trial (RCT) revealed that implementation of the Teach-Back educational intervention was associated with statistically significant improvements in self-care adherence (*P* < 0.05) among heart failure patients during the post-discharge period (12). The Teach-back method, requiring patients to paraphrase medical instructions, ensures comprehension, and replaces traditional unidirectional education with interactive engagement. Furthermore, Wang et al. (13) demonstrated that integrating the Teach-back method with video education in home care instruction significantly improved self-care capacity and reduced caregiver burden among stroke patients. These findings suggest that combining innovative strategies with multidisciplinary team (MDT) collaboration could further enhance patients' mastery of rehabilitation skills through integrated professional knowledge delivery and personalized education.

Health-promoting behaviors—encompassing health responsibility, exercise, self-actualization, nutrition, interpersonal relationships, and stress management—were evaluated using the HPLP-II scale (14). At 2-month follow-up, the observation group exhibited significantly higher HPLP-II scores than controls (*P* < 0.05), confirming the intervention's efficacy in enhancing health behaviors. Research on total knee arthroplasty has demonstrated that health-promoting lifestyles significantly contribute to improved quality of life, prevention of disease recurrence, and reduction of post-surgical complications (15). Personalized education aligned with patient-specific needs, coupled with post-discharge continuity of care through regular follow-ups and guidance, reinforced self-management capabilities and sustained rehabilitation adherence, thereby consolidating long-term behavioral improvements.

Psychological status, assessed via STAI scores, revealed significant reductions in anxiety levels within the observation group at 2-week post-intervention (*P* < 0.05). Prolonged activity restriction may adversely affect patients' psychological status, potentially leading to increased dependency on healthcare providers among certain patient populations (16). The Teach-back method fostered cognitive clarity and achievement motivation through knowledge verification, while continuity of care provided sustained emotional support. By addressing psychological needs via active listening and encouragement, this dual approach cultivated patient confidence and positive engagement with rehabilitation challenges. Furthermore, self-actualization and stress management components of health-promoting behaviors may enhance psychological wellbeing by reducing anxiety and depression, thereby facilitating comprehensive rehabilitation (17).

Notably, the observation group demonstrated lower incidence rates of deep vein thrombosis, joint stiffness, pressure injuries, and infections compared to controls. These complications, typically associated with delayed mobilization and restricted postoperative activity in conventional care, were mitigated through structured Teach-back-enhanced preoperative education and continuity of care. Pain remains the primary obstacle to physical activity, particularly in patients with joint injuries, which further limits beneficial postoperative exercise (18). The real-time correction of cognitive misconceptions during Teach-back sessions optimized treatment compliance, while post-discharge continuous monitoring ensured sustained adherence to personalized rehabilitation protocols, thereby effectively reducing complication risks.

In conclusion, the integration of Teach-back health education with continuity of care demonstrates significant advantages in knee training injury rehabilitation. By enhancing knowledge retention, self-care capacity, early problem identification, and clinician-patient trust, this model reduces postoperative complications, improves psychological wellbeing, and elevates health-promoting behaviors, ultimately accelerating functional recovery and preserving military operational readiness. This innovative approach provides a novel framework for rehabilitation nursing in training-related injuries, with broad clinical applicability.

## Data Availability

The raw data supporting the conclusions of this article will be made available by the authors, without undue reservation.
