# Supplementary material for: Application of Teach-back health education combined with continuity care in patients with knee joint training injuries: an analysis of clinical effects
Source: Front Public Health. 2025 May 21;13:1577538. doi: 10.3389/fpubh.2025.1577538 (PMC12133758; doi:10.3389/fpubh.2025.1577538)
Supplement: Supplementary file 1 [file Table_1.docx]

**Supplementary methods**

1. Meniscus injuries rehabilitation protocol:
2. Muscle Strength Training

Postoperative Day 0: Initiate ankle pumps, straight leg raises, and calf lifts with knee brace locked at 0-30° flexion. Adjust to 0°-0° if pain occurs.

Postoperative Days 2-6: Perform patellar mobilization (bidirectional gliding, twice daily); Implement knee compression exercises: Place 5cm pillow under ankle, alternate knee elevation (3cm) and depression (30min/session, tid).

Postoperative Week 2: Progress to quadriceps/hamstring strengthening: Ankle pumps with resistance bands; Weighted straight leg raises (gradual load increase), 30min/session, tid

(2) Range of Motion (ROM) Training

Week 1: Passive flexion to 90°

Week 2: 100° passive flexion

Week 3: 110° passive flexion

Week 4: 120° passive flexion

Weeks 5-7: Maintain ≤120° passive flexion

Week 8+: Progress to active flexion beyond 120°

(3) Weight-Bearing Progression

Weeks 0-2: Non-weight bearing (extend to 4 weeks for body tears)

Week 3: 25% partial weight-bearing (toe touch)

Week 4: 50% weight-bearing (forefoot contact)

1. Ligament reconstruction rehabilitation protocol:

(1) Phase I (0-2 Weeks)

Day 0: Ankle pumps, quadriceps isometrics

Day 1: Crutch-assisted ambulation (non-weight bearing)

Day 2: Add lateral/back leg lifts

Day 3: Begin protected weight shifting

Day 4: Single-leg stance training

Day 5: Active ROM (70°-80° flexion)

Weeks 1-2: Achieve 90° active flexion

(2) Phase II (2-4 Weeks)

Week 2: Passive flexion to 100°, wean to single crutch

Week 3: Advance to 110° flexion, initiate open-chain exercises

Week 4: Full weight-bearing, begin step-ups

(3) Phase III (5 Weeks-3 Months)

Week 5: Stationary cycling (no resistance)

Weeks 8-10: Progress to deep squatting

Months 2-3: Implement proprioceptive training

(4) Phase IV (4-6 Months)

Initiate: Lateral bounding; Low-impact jogging; Swimming (avoid breaststroke)

(5) Phase V (7-12 Months)

Implement: Agility drills; Plyometrics; Sport-specific training
