# Supplementary material for: Application of Teach-back health education combined with continuity care in patients with knee joint training injuries: an analysis of clinical effects
Source: Front Public Health. 2025 May 21;13:1577538. doi: 10.3389/fpubh.2025.1577538 (PMC12133758; doi:10.3389/fpubh.2025.1577538)
Supplement: Supplementary file 2 [file Table_2.docx]

Supplementary Table 1. The knowledge assessment questionnaire on rehabilitation care for patients with knee training injuries.

| **Time Period** | **Health Education Content** | **Education Time** | **Educator** | **Mastery Status (Yes/No)** |
| --- | --- | --- | --- | --- |
| ****Preoperative**** | • Mechanisms of meniscus and anterior cruciate ligament injuries • Surgical procedures and rehabilitation |  |  |  |
|  | • Preoperative fasting duration |  |  |  |
|  | • Psychological care methods |  |  |  |
|  | • Bed rest duration and clinical significance |  |  |  |
|  | • Bowel management strategies |  |  |  |
|  | • Respiratory function training methods |  |  |  |
| ****Postoperative**** | • Postoperative dietary interventions |  |  |  |
|  | • Postoperative pain assessment methods (NRS/FACES scale) |  |  |  |
|  | • Non-pharmacological pain relief techniques (e.g., cold therapy, positioning) |  |  |  |
|  | • Anxiety and stress relief techniques |  |  |  |
|  | • Early limb mobilization protocols during bed rest |  |  |  |
|  | • Criteria for initiating ambulation and gait training |  |  |  |
|  | • Respiratory function retraining |  |  |  |
| ****Rehabilitation**** | • Postoperative muscle strength training methods (e.g., isometric exercises) |  |  |  |
|  | • Progressive joint mobility training protocols |  |  |  |
|  | • Weight-bearing progression criteria |  |  |  |
| ****Discharge & Follow-up**** | • Post-discharge precautions (e.g., wound care, activity restrictions) |  |  |  |
|  | • Medication administration and dietary guidelines |  |  |  |
|  | • Schedule for clinical re-evaluation |  |  |  |
|  | • Home-based functional exercise protocols |  |  |  |
|  | • Ability to demonstrate postoperative functional exercises via video |  |  |  |
